# Supplementary material for: Heat adaptation of phage T7 under an extended genetic code
Source: Virus Evol. 2021 Dec 1;7(2):veab100. doi: 10.1093/ve/veab100 (PMC8923235; doi:10.1093/ve/veab100)
Supplement: veab100_Supp [file veab100_supp.zip › Sequencing_Analysis.zip!/Sequencing_Analysis/S1/evidence/JC_SIDE_1_40.html]

BRESEQ :: Evidence


| New junction evidence | | | | | | | | | | | |
| --- | --- | --- | --- | --- | --- | --- | --- | --- | --- | --- | --- |
|  | | seq id | position | reads (cov) | reads (cov) | score | skew | freq | annotation | gene | product |
| \* | ? | NC\_001604.1 | = 281 | 3834 (1.030) | 908 (0.230) +TAAAGACCAGACC | 18/508 | 36.9 | 18.1% | intergenic (–/–) | *–/–* | –/– |
|  | ? | NC\_001604.1 | 282 = | 4288 (1.020) |  |  |  |  | intergenic (–/–) | *–/–* | –/– |
| Rejected: Coverage evenness skew score above cutoff. | | | | | | | | | | | |
| Rejected: Frequency below/above cutoff threshold. | | | | | | | | | | | |

Only 100 of 5199 total aligned reads displayed.  

`‑‑‑‑‑‑‑‑‑‑‑‑‑TCTCACAGTGTACGGACCTAAAGTTCCCCCATAGGGGGTACCTAAAGCCCAGCCAATCACCTAAAGTCAACCTTCGGTTGACCTTGAGGGTTCCCTAAGGGTTGGGGATGACCCTTGGGTTTGTCTTTGGGTGTTACCTTGAGTGTCTCTCTGTGTCCCTATCTGTTACAGTCTCCTAAAGTATCCTCCTAAAGTCACCTCCTAACGTCCATCCTAAAGCCAACACCTAAAGCCTACACCTAAAGACCCATCAAGTCAACGCCTATCTTAAAGTTTAAACATAAAGACCAGACCTAAAGACCAGACCTAAAGACACTACATAAAGACCAGACCTAAAGACGCCTTGTTGTTAGCCATAAAGTGATAACCTTTAATCATTGTCTTTATTAATACAACTCACTATAAGGAGAGACAACTTAAAGAGACTTAAAAGATTAATTTAAAATTTATCAAAAAGAGTATTGACTTAAAGTCTAACCTATAGGATACTTACAGCCATCGAGAGGGACACGGCGAATAGCCATCCCAATCGACACCGGGGTCAACCGGATAAGTAGACAGCCTGATAAGTCGCACGAAAA  >  NC_001604.1/1‑581`  

                                                                                                                                                                                                                                                                                                     `|`                                                                                                                                                                                                                                                                                                              
`gagatgtgtataagagacaggtggACGGACCTAAAGTTACCCCATAGGGGGTACCTAAAGCCCAGCCAATCACCTAAAGTCAACCTTGGGTTGACATGGAGGGTTCCCTAAGGGTTGGGGATGACCCTTGGGTTTGTCTTTGGGTGTTACCTTGAGTGTCTCTCTGTGTCCCTATCTGTTACAGTCGCCTAAAGTATCCTCCTAAAGTCACCTCCTAACGTCCATCCTAAAGCCAACACCTAAAGCCTACACCTAAAGACCCATCAAGTCAACGCCTATCTTAAAGTTTAAACAtaaagac                                                                                                                                                                                                                                                                                                       <  1:478042/281‑1 (MQ=25)`  
           `tgtgtataagagacaggACCTAAAGTTCCCCCATAGGGGGTACCTAAAGCCCAGCCAATCACCTAAAGTCAACCTTCGGTTGACCTTGAGGGTTCCCTAAGGGTTGGGGATGACCCTTGGGTTTGTCTTTGGGTGTTACCTTGAGTGTCTCTCTGTGTCCCTATCTGTTACAGTCTCCTAAAGTATCCTCCTAAAGTCACCTCCTAACGTCCATCCTAAAGCCAACACCTAAAGCCTACACCTAAAGACCCATCAAGTCAACGCCTATCTTAAAGTTTAAACGtaaagaccagacctaaag                                                                                                                                                                                                                                                                                            <  1:208703/286‑1 (MQ=255)`  
              `ttttatggaacaggACCTAAATGTCCCCCCAAGGGGGTACCGAAAGCCCAGCCAAACCCCTAAAGTCAACCTTCGGTTGACCTTGAGGGTTCCCTAAGGGTTGGGGATGACCCTTGGGTTTTTCTTTGGGTGTTACCTTGAGTGTCTCTCTGTGTCCCTATCTGTTACAGTCTCCTAAAGTATCCTCCTAAAGTCACCTCCTAACGTCCATCCTAAAGCCAACACCTAAAGCCTACACCTAAAGACCCATCAAGTCAACGCCTATCTTAAAGTTTAAACAtaaagcccagacctaaagacc                                                                                                                                                                                                                                                                                         <  1:70038/289‑1 (MQ=25)`  
              `agacaggtgtACGGACCTAAAGTTCCCCCATAGGGGGTACCTAAAGCCCAGCCAATCACCTAAAGTCAACCTTCGGTTGACCTTGAGGGTTCCCTAAGGGTTGGGGATGACCCTTGGGTTTGTCTTTGGGTGTTACCTTGAGTGTCTCTCTGTGTCCCTATCTGTTACAGTCTCCTAAAGTATCCTCCTAAAGTCACCTCCTAACGTCCATCCTAAAGCCAACACCTAAAGCCTACACCTAAAGACCCATCAAGTCAACGCCTATCTTAAAGTTTAAACAtaaagaccagacctaaagacc                                                                                                                                                                                                                                                                                         <  1:100028/295‑1 (MQ=255)`  
                    `gtgtACGGACCTAAAGTTCTCCCATAGGGGGTACCTAAAGCCCAGCCAATCACCTAAAGTCAACCTTCGGTTGACCTTGCGGGTTCCCTAAGGGTTGGGGATGACCCTTGGGTTTGTCTTTGGGTGTTACCTTGAGTGTCTCTCTGTGTCCCTATCTGTTACAGTCTCCTAAAGTATCCTCCTAAAGTCACCTCCTAACGTCCATCCTAAAGCCAACACCTAAAGCCTACCCCTAAAGACCCATCAAGTCAACGCCTATCTTAAAGTTTAAACAtaaagacctgtatcttatacacctctc                                                                                                                                                                                                                                                                                   >  1:502895/1‑282 (MQ=255)`  
                    `gtgtACGGACCTAAAGTTCCCCCATAGGGGGTACCTAAAGCCCAGCCAATCACCTAAAGTCAACCTTCGGTTGACCTTGAGGGTTCCCTAAGGGTTGGGGATGACCCTTGGGTTTGTCTTTGGGTGTTACCTTGAGTGTCTCTCTGTTTCCCTATCTGTTACAGTCTCCTAAAGTATCCTCCTAAAGTCACCTCCTAACGTCCATCCTAAAGCCAACCCCTAAAGCCTACACCTAAAGACCCATCAAGTCAACGCCTATCTTAAAGTTTAAACAtaaagaccagacctaaagctgtctctt                                                                                                                                                                                                                                                                                   >  1:607477/1‑292 (MQ=255)`  
                    `gtgtACGGACCTAAAGTTCCCCCATAGGGGGTACCTAAAGCCCAGCCAATCACCTAAAGTCAACCTTCGGTTGACCTTGAGGGTTCCCTAAGGGTTGGGGATGACCCTTGGGTTTGTCTTTGGGTGTTACCTTGAGTGTCTCTCTGTGTCCCTATCTGTTACAGTCTCCTAAAGTATCCTCCTAAAGTCACCTCCTAACGTCCATCCTAAAGCCAACACCTAAAGCCTACACCTAAAGACCCATCAAGTCAACGCCTATCTTAAAGTTTAAACATaaagcccagacctaaagaccagacct                                                                                                                                                                                                                                                                                   >  1:496185/1‑301 (MQ=255)`  
                    `gtgtACGGACCTAAAGTTCCCCCATAGGGGGTACCTAAAGCCCAGCCAATCACCTAAAGTCAACCTTCGGTTGACCTTGAGGGTTCCCTAAGGGTTGGGGATGACCCTTGGGTTTGTCTTTGGGTGTTACCTTGAGTGTCTCTCTGTGTCCCTATCTGTTACAGTCTCCTAAAGTATCCTCCTAAAGTCACCTCCTAACGTCCATCCTAAAGCCAACACCTAAAGCCTACACCTAAAGACCCATCAAGTCAACGCCTATCTTAAAGTTTAAACATaaagaccagacctaaagaccagacct                                                                                                                                                                                                                                                                                   >  1:593886/1‑301 (MQ=255)`  
                    `gtgtACGGACCTAAAGTTCCCCCATAGGGGGTACCTAAAGCCCAGCCAATCACCTAAAGTCAACCTTCGGTTGACCTTGAGGGTTCCCTAAGGGTTGGGGATGACCCTTGGGTTTGTCTTTGGGTGTTACCTTGAGTGTCTCTCTGTGTCCCTATCTGTTACAGTCTCCTAAAGTATCCTCCTAAAGTCACCTCCTAACGTCCATCCTAAAGCCAACACCTAAAGCCTACACCTAAAGACCCATCAAGTCAACGCCTATCTTAAAGTTTAAACATaaagaacagacctaaagaccagacct                                                                                                                                                                                                                                                                                   >  1:27389/1‑301 (MQ=255)`  
                    `gtgtACGGACCTAAAGTTCCCCCATAGGGGGTACCTAAAGCCCAGCCAATCACCTAAAGTCAACCTTCGGTTGACCTTGAGGGTTCCCTAAGGGTTGGGGATGACCCTTGGGTTTGTCTTTGGGTGTTACCTTGAGTGTCTCTCTGTGTCCCTATCTGTTACAGTCTCCTAAAGTATCCTCCTAAAGTCACCTCCTAACGTCCATCCTAAAGCCAACACCTAAAGCCTACACCTAAAGACCCATCAAGTCAACGCCTATATTAAAGTTTAAACAtaaagacctgtctcttatacacatctc                                                                                                                                                                                                                                                                                   >  1:534372/1‑282 (MQ=255)`  
                    `gtgtACGGACCTAAAGTTCCCCCATAGGGGGTACCTAAAGCCCAGCCAATCACCTAAAGTCAACCTTCGGTTGACCTTGAGGGTTCCCTAAGGGTTGGGGATGACCCTTGGGTTTGTCTTTGGGTGTTACCTTGAGTATCTCTCTGTGTCCCTATCTGTTACAGTCTCCTAAAGTATCCTCCTAAAGTCACCTCCTAACGTCCATCCTAAAGCCAACACCTAAAGCCTACACCTAAAGACCCATCAAGTCAACGCCTATCTTAAAGTTTAAACATaaagaccagacctaaagaccagacct                                                                                                                                                                                                                                                                                   >  1:286514/1‑301 (MQ=255)`  
                      `gtACGGACCTAAAGTTCCCCCATAGGGGGTACCTAAAGCCCAGCCAATCACCTAAAGTCAACCTTCGGTTGACCTTGAGGGTTCCCTAAGGGTTGGGGATGACCCTTGGGTTTGTCTTTGGGTGTTACCTTGAGTGTCTCTCTGTGTCCCTATCTGTTACAGTCTCCTAAAGTATCCTCCTTAAGTCCCCTCCTAACGTCCATCCTAAAGCCAACACCTAAAGCCTACACCTAAAGACCCATCAAGTCAACGCCTATCTTAAAGTTTAAACATAAagaccagacctaaagaccagacctaa                                                                                                                                                                                                                                                                                 >  1:334633/1‑301 (MQ=255)`  
                      `gtACGGACCTAAAGTTCCCCCATAGGGGGTACCTAAAGCCCAGCCAATCACCTAAAGTCAACCTTCGGTTGACCTTGAGGGTTCCCTAAGGGTTGGGGATGACCCTTGGGTTTGTCTTTGGGTGTTACCTTGAGTGTCTCTCTGTGTCCCTATCTGTTACAGTCTCCTAAAGTATCCTCCTAAAGTCACCTCCTAACGTCCATCCTAAAGCCAACACCTAAAGCCTACACCTACAGACCCATCAAGTCAACGCCTATCTTAAAGTTTAAACAtaaagaccagacctaaagaccctgtctct                                                                                                                                                                                                                                                                                 >  1:249840/1‑293 (MQ=255)`  
                      `gtACGGACCTAAAGTTCCCCCATAGGGGGTACCTAAAGCCCAGCCAATCACCTAAAGTCAACCTTCGGTTGACCTTGAGGGTTCCCTAAGGGTTGGGGATGACCCTTGGGTTTGTCTTTGGGTGTTACCTTGAGTGTCTCTCTGTGTCCCTATCTGTTACAGTCTCCTAAAGTATCCTCCTAAAGTCACCTCCTAACGTCCATCCTAAAGCCAACACCTAAAGCCTACACCTAAAGACCCATCAAGTCAACGCCTATCTTAAAGTTTAACCATAAagaccagacctaaagaccagaactaa                                                                                                                                                                                                                                                                                 >  1:110899/1‑301 (MQ=255)`  
                      `gtACGGACCTAAAGTTCCCCCATAGGGGGTACCTAAAGCCCAGCCAATCACCTAAAGTCAACCTTCGGTTGACCTTGAGGGTTCCCTAAGGGTTGGGGATGACCCTTGGGTTTGTCTTTGGGTGTTACCTTGAGTGTCTCTCTGTGTCCCTATCTGTTACAGTCTCCTAAAGTATCCTCCTAAAGTCACCTCCTAACGTCCATCCTAAAGCCAAAACCTAAAGCCTACACCTAAAGACCCATCTAGTCAACGCCTATCTTAAAGTTTAAACAtaacgaccagacctaaagaagtctcttat                                                                                                                                                                                                                                                                                 >  1:18502/1‑291 (MQ=255)`  
                      `gtACGGACCTAAAGTTCCACCATAGGGGGTACCTAAAGCCCAGCCAATCACCTAAAGTCAACCTTCGGTTGACCTTGAGGGTTCCCTAAGGGTTGGGGATGACCCTTGGGTTTGTCTTTGGGTGTTACCTTGAGTGTCTCTCTGTGTCCCTATCTGTTACAGTCTCCTAAAGTATCCTCCTAAAGTCACCTCCTAACGTCCATCCTAAAGCCAACACCTACAGCCTACACCTAAAGACCCATCAAGTCAACGCCTATCTTACAGTTTAAACAtcaagaccagccctaaagcccctttctct                                                                                                                                                                                                                                                                                 >  1:365890/1‑293 (MQ=255)`  
                           `gACCTAAAGTTCCCCCATAGGGGGTACCTAAAGCCCAGCCAATCACCTAAAGTCAACCTTCGGTTGACCTTGAGGGTTCCCTAAGGGTTGGGGATTACCCTTGGGTTTGTCTTTGGGTGTTACCTTGAGTGTCTCTCTGTGTCCCTATCTGTTACAGTCTCCTAAAGTATCCTCCTAAAGTCACCTCCTAACGTCCATCCTAAAGCCAACACCTAAAGCCTACACCTAAAGACCAATCAAGTCAACGCCTATCTTAAAGTTTAAACAtaaagaccagacctaaagctgtctcttatacaca                                                                                                                                                                                                                                                                            >  1:400472/1‑285 (MQ=255)`  
                           `gACCTAAAGTTCCCCCATAGGGGGTACCTAAAGCCCAGCCAATCACCTAAAGTCAACCTTCGGTTGACCTTGAGGGTTCCCTAAGGGTTGGGGATGACCCTTGGGTTTGTCTTTGGGTGTTACCTTGAGTGTCTCTCTGTGTCCCTATCTGTTACAGTCTCCTAAAGTATCCTCCTAAAGTCACCTCCTAACGTCCATCCTAAAGCCAACACCTAAAGCCTACACCTAAAGACCCATCAAGTCAACGCCTATCTTAAAGTTTAAACCTAAAGAccagacctaaagaccagacctcaagaaa                                                                                                                                                                                                                                                                            >  1:315906/1‑299 (MQ=255)`  
                                 `gtataagagacaggggggTACATAAAGCCAAGCCAATCACCTAAAGTCAACCTTCGGTTGGCCTTGAGGGTTCCCGAATGGTTGGGGATGACCCTTGGGTTTGTCTTTGGGTGTTACCTTGAGTGTCTCTCTGTGTCCCTATCTGTTACAGTCTCCTAAAGTATCCTCCTAAATTCACCTCCTAACGTCCATCCTAAAGCCAACACCTAAAGCCTACACCTAAAGACCCATCAAGTCAACGCCTATCTTAAAGTTTAAACATAAATACCAGACCTAAAGACCAGACCTAAAGACACTACAt                                                                                                                                                                                                                                                                      <  1:148867/288‑1 (MQ=37)`  
                                   `gTTCCCCCATAGGGGGTACCTAAAGCCCAGCCAATCACCTAAAGTCAACCTTCGGTTGACCTTGAGGGTTCCCTAAGGGTTGGGGATGACCCCTGGGTTTGTCTTTGGGTGTTACCTTGAGTGTCTCTCTGTGTCCCTATCTGTTACAGTCTCCTAAAGTATCCTCCTACAGTCCCCTCCTTACGTCCATTCTAAAGCAAACACCTAATGCCCACACCTAAAGACCCATCAAGTCAACGCATATCTTTAAGTTTAAACATATAGACCAGACTCAAAGACCAGTCCACAAGCCCCTACAtca                                                                                                                                                                                                                                                                    >  1:50763/1‑299 (MQ=21)`  
                                     `acagccATAGGGGACACCTAAAGCCCAGACAATCACCTAAAGTCAACCTTCGGTTGAACTGAAGGGGTCCCTAAGGGTTGGGGATGACAAGTGGGTTAGTCTTTGGGTGTTACCTTGAGTGTCTCTCTGTGTCCCTGTCTGTTACAGTCTCCTAAAGTATCCTCCTAAAGTCACCACCTAACGTCCATCCTAAAGCCAACACATAAAGCCTACACCTAAAGACCCATCAAGTCAACGCCTATCGTAAAGTTTAAACATAAAGACCAGGCCTAAAGACCAGACCTAAAGACACTACATAAag                                                                                                                                                                                                                                                                  <  1:172298/297‑1 (MQ=37)`  
                                       `gcccATAGGGGGTACCTAAAGCCCAGCCAATCACCTAAAGTCAACCTTCGGGTGACCGTGAGGGTTCCCTAAGGGTTGGGGATGACCCTTGGGTTTGTCTTTGGGTGTTACCTTGAGTGTCTCTCTGTGTCCCTATCTGTTACAGTCTCCTAAAGTATCCTCCTAAAGTCACTTCCTAACGTCCATCCTAAAGCCAACACCTAAAGCCTACACCTAAAGACCCATCAAGTCAACGCCTATCTTAAAGTTTAAACATAAAGACCAGACCTAAAGACCAGACCTAAAGACACTACATAAagac                                                                                                                                                                                                                                                                <  1:268426/300‑1 (MQ=255)`  
                                         `ccATAGGGGGTACCTAAAGCCCAGCCAATCACCTAAAGTCAACCTTCGGTTGACCTTGAGGGTTCCCTAAGGGTTGGGGATGACCCTTGGGTTTGTCTTTGGGTGTTACCTTGAGTGTCTCTCTGTGTCCCTATCTGTTACAGTCTCCTAAAGTATCCTCCTAAAGTCACCTCCTAACGTCCATCCTAAAGCCAACACCTAAAGCCTACACCTAAAGACCCATCAAGTCAACGCCTATCTTAAAGTTTAAACATAAAGACCAGACCTAAAGACCAGACCTAAAGACACTACATAAagacct                                                                                                                                                                                                                                                              >  1:214368/1‑300 (MQ=255)`  
                                              `gggggTACCTAAAGCCCAGCCAATCACCTAAAGTCAACCTTCGGTTGACCTTGAGGGTTCCCTAAGGGTTGGGGATGACCCTTGGGTTTGTCTTTGGGTGTTACCTTGAGTGTCTCTCTGTGTCCCTATCTGTTACAGTCTCCTAAAGTATCCTCCTAAAGTCACCTCCTCACGTCCATCCTAAAGCCAACACCTAAAGCCTACACCTAAAGACCCATCCAGTCAACGCCTATCTTAAAGTTTAAACATAAAGACCAGACCTAAAGGCCAGACCTAAAGACACTACATaaactgtctctca                                                                                                                                                                                                                                                         >  1:430011/1‑291 (MQ=255)`  
                                                  `aagagacaggCCCAGCCAATCACCTAAAGTCAACCTTCGGTTGACCTTGAGGGTTCCCTAAGGGTTGGGGATGACCCTTGGGTTTGTCTTTGGGTGTTACCTTGAGTGTCTCTCTGTGTCCCTATCTGTTACAGTCTCCTAAAGTATCCTCCTAAGGTCACCTCCTAACGTCCATCCTAAAGCCAACACCTAAAGCCTACACCTAAAGACCCATCAAGTCAACGCCTATCCTAAAGTTTAAACATAAAGACCAGACCTAAAGACCAGACCTAAAGACACTACATAAAGACCAGACCTAAAg                                                                                                                                                                                                                                                     <  1:340875/292‑1 (MQ=255)`  
                                                    `aCCTAAAGCCCAGCCAATCACCTAAAGTCAACCTTCGGTTGACCTTGAGGGTTCCCTAAGGGTTGGGGATGACCCTTGGGTTTGTCTTTGGGTGTTACCTTGAGTGTCTCTCTGTGTCCCTATCTGTTACAGTCTCCTAAAGTATCCTCCTAAAGTCACCTCCTAACGTCCATCCTAAAGCCAACACCTAAAGCCTACACCTAAAGACCCATCAAGTCAACGCCTATCTTAAAGTTTAAACATAAAGACCAGACCTAAAGACCAGACCTAAAGACACTACATAAAGACCAGACCTAAAGAc                                                                                                                                                                                                                                                   >  1:291432/1‑301 (MQ=255)`  
                                                              `cAGCCAATCACCTAAAGTCAACCTTCGGTTGACCTTGAGGGTTCCCTAAGGGTTGGGGATGACCCTTGGGTTTGTCTTTGGGTGTTACATTGAGTGTCTCTCTGTGTCCCTATCTGTTACAGTCTCCTAAAGTATCCTCCTAAAGTCACCTCCTAACGTCCATCCTAAAGCCTACACCTAAAGCCTACACCTAAAGACCCATCAAGTCAACGCCTATCTTAAAGTTTAAACATAAAGACCAGACCTAAAGACCAGACCTAAAGACACTACATAAAGACCAGACCTAAAGACGCCTtgttgt                                                                                                                                                                                                                                         <  1:15501/301‑1 (MQ=255)`  
                                                                       `aCCTAAAGTCAACCTTCGGTTGACCTTGAGGGTTCCCTAAGGGTTGGGGATGACCCTTGGGTTTGTCTTTGGGTGTTACCTTGAGTGTCTCTCTGTGTCCCTATCTGTTACAGTCTCCTAAAGTATCCTCCTAAAGTCACCTCCTAACGTCCATCCTAAAGCCAACACCTAAAGCCTACACCTAAAGACCCATCAAGTCAACGCCTATCTTAAAGTTTAAACATAAAGACCATACCTAAAGACCAGACCTAAAGACACTACATAAAGACCAGACCTAAAGACGCCttgttgctgtctctta                                                                                                                                                                                                                                >  1:389666/1‑291 (MQ=255)`  
                                                                        `ccTAAAGTCAACCTTCGGTTGACCTTGAGGGTTCCCTAAGGGTTGGGGATGACCCTTGGGTTTGTCTTTGGGTGTTACCTTGAGTGTCTCTCTGTGTCCCTATCTGTTACAGTCTCCTAAAGTATCCTCCTAAAGTCACCTCCTAACGTCCATCCTAAAGCCAACACCTAAAGCCTACACCTAAAGACCCATCAAGTCAACGCCTATCTTAAAGTTTAAACATAAAGACCAGACCTAAAGACCAGACCTAAAGACACTACATAAAGACCAGACCTAAAGACGCCTTGTTTTTAGCCATaaa                                                                                                                                                                                                                               >  1:236053/1‑301 (MQ=255)`  
                                                                         `cTAAAGTCAAACTTCGGTTGAACTTGAGGGTTCCCTAAGGTGTGGGGATGACCCTTGGGTTTGTCTGGGGGTTTTAACTTGAGTGTCTCTCTGTGTACACATATGTTACAGTCTCCTAAAGTATACTCATAAAGTCACCTCATAACGTCCATCCTAAAGCCAAAACCTAAAGCCTACACCTAAAGACCCATCAAGTCAACGCATATCTTAAAGTTTAAACATAAAGACCAGACCTAAAGACCAGACCTAAAGACACTACATAAAGACCAGACCTAAAGACGCCTTGTTGTTAGCCATAAAg                                                                                                                                                                                                                              <  1:619311/301‑1 (MQ=255)`  
                                                                           `aaaGTCAACCTTCGGTTGACCTTGAGGGTTCCCTAAGGGTTGGGGATGACCCTTGGGTTTGTCTTTGGGTGTTACCTTGAGTGTCTCTCTGTGTCCCTATCTGTTACAGTCTCCTAAAGTATCCTCCTAAAGTCACCTCCTAACGTCCATCCTAAAGCCAACACCTAAAGCCTACCCCTAAAGACCCATCAAGTCAACGCCTATCTTAAAGTTTAAACATACAGACCAGACCTAAAGCCCAGACCTAAAGACACTACATAAAGATCAGACCTAACGACACCTTATTTTTAGCCATaaattt                                                                                                                                                                                                                            >  1:346945/1‑298 (MQ=255)`  
                                                                                 `cgtgggctcggagatgtgtataagagacaggggTTGGGGATGACCCTTGGGTTTGTCTTTGGGTGTTACCTTGAGTGTCTCTCTGTGTCCCTATCTGTTACAGTCTCCTAAAGTATCCTCCTAAAGTCACCTCCTAACGTCCATCCTAAAGCCAACACCTAAAGCCTACACCTAAAGACCCATCAAGTTAACGCCTATCTTAAAGTTTAAACATAAAGACCAGACCTAAAGACCAGACCTAAAGACACTACATAAAGACCAGACCTAAAGACGCCTTGTTGTTAGCCATAAAGTGATAAcc                                                                                                                                                                                                                      <  1:600601/271‑1 (MQ=255)`  
                                                                                        `gggtGACCTGGAGGGGTCCCTAAGGGTTGGGGATGACCCTTGGGTTTGTCTTTGGGTGTTACCTTGAGTGTCTCTCTGTGTCCCTATCTGTTACAGTCTCCTAAAGTATCCTCCTAAAGTCACCTCCTAACGTCCATCCTAAAGCCAACACCTAAAGCCTACACCTAAAGACCCATCAAGTCAACGCCTATCTTAAAGTTTAAACATAAAGACCAGACCTAAAGACCAGACCTAAAGACACTACATAAAGACCAGACCTAAAGACGCCTTGTTGTTAGCCATAAAGTGATAACCTTTAATc                                                                                                                                                                                                               <  1:40609/298‑1 (MQ=255)`  
                                                                                        `ggTTGACCTTGAGGGTTCCCTAAGGGTTGGGGATGACCCTTGGGTTTGTCTTTGGGTGTTACCTTGAGTGTCTCTCTGTGTCCCTATCTGTTACAGTCTCCTAAAGTATCCTCCTAAAGTCACCTCCTAACGTCCATCCTAAAGCCAACACCTAAAGCCTACACCTAAAGACCCATCAAGTCAACGCCTATCTTAAAGTTTAAACATAAAGACCAGACCTAAAAACCAGACCTAAAGACACTACATAAAGACCAGACCTAAAGACGCCTTGTTGTTAGCCATAAAGTGATAACCTTTAATc                                                                                                                                                                                                               <  1:668091/301‑1 (MQ=255)`  
                                                                                        `gacagACCTTGAGGGTTCCCTAAGGGTTGGGGATGACCCGTTGGTTTGTCTTTGGGTGTTACCTTGAGTGTCTCTCTGTGTCCCTATCTGTTACAGTCTCCTAAAGTATCCTCCTAAAGTCACCTCCTAACGTCCATCCTAAAGCCAACACCTAAAGCCTACACCTAAAGACCCATCAAGTCAACGCCTATCTTAAAGTTTAAACATAAAGACCAGACCTAAAGACCAGACCTAAAGACACTACATAAAGACCAGACCTAAAGACGCCTTGTTGTTAGCCATAAAGTGATAACCTTTAATc                                                                                                                                                                                                               <  1:302976/297‑1 (MQ=255)`  
                                                                                        `atgtgtataagagacagcccTAAGGGTTGGGGATTACCCTTGTGTTTGTTTTTTGGTGTTACCTTGAGTGTCTCTCTGTGTCCCTATCTGTTACAGTCTCCTAAAGTATCCTCCTAAAGTCACCTCCTAACGTCCATCCTAAAGCCAACACCTAAAGCCTACACCTAAAGACACATCAAGTCAACGCCTATCTTAAAGTTTAAACATAAAGACCAGACCTAAAGACCAGACCTAAAGACACTACATAAAGACCAGACCTAAAGACGCCTTGTTGTTAGCCATAAAGTGATAACCTTTAATc                                                                                                                                                                                                               <  1:328633/284‑1 (MQ=255)`  
                                                                                              `ccTTGAGGGTTCCCTAAGGGTTGGGGATGACCCTTGGGTTTGTCTTTGGGTGTTACCTTGAGTGTCTCTCTGTGTCCCTATCTGTTACAGTCTCCTAAAGTATCCTCCTAAAGTCACCTCCTAACGTCCATCCTAAAGCCAACACCTAAAGCCTACACCTAAAGACCCATCAAGTCAACGCCTATCTTAAAGTTTAAACATAAAGACAAGACCTAAAGACCAGACCTAAAGACACTACATAAAGACCAGACCTAAAGACGCCTTGTCGTTAGCCATAAAGTGATAACATTTAATcctgtcc                                                                                                                                                                                                         >  1:539440/1‑295 (MQ=255)`  
                                                                                              `ccTTGAGGGTTCCCTAAGGGTTGGGGATGACCCTTGGGTTTGTCTTTGGGTGTTACCTTGAGTGTCTCTCTGTGTCCCTATCTGTTACAGTCTCCTAAAGTATCCCCCTAAAGTCACCTCCTAACGTCCATCCTAAAGCCAACACCT‑AAGCCTACACCTAAAGACCCATCAAGTCAACGCCTATCTTAAAGTTTAAACATAAAGACCAGACCTAAAGACCAGACCTAAAGACACTACATAAAGACCAGACCTAAAGACGCCTTGTTGTTAGCCATAAAGTGATAACCTTTAATCATTGTCt                                                                                                                                                                                                        >  1:220899/1‑301 (MQ=255)`  
                                                                                               `tatgagacagcccTAAGTGTTGGGGATGACCCTTGGGTTTGTCTTTGGGTGTTACCTTGAGTGTCTCTCTGTGTCCCTATCTGTTACAGTCTCCTAAAGTATCCTCCTAAAGTCACCTCCTAACGTCCATCCTAAAGCCAACACCTAAAGCCTACACCTAAAGACCCATCAAGTCAACGCCTATCTTAAAGTTTAAACATAAAGACCAGACCTAAAGACCAGACCTAAAGACACTACATAAAGACCAGACCTAAAGACGCCTTGTTGTTAGCCATAAAGTGATAACCTTTAATCATTGTCt                                                                                                                                                                                                        <  1:546830/291‑1 (MQ=255)`  
                                                                                                    `gggTTCCCTAAGGGTTGGGGATGACCCTTGGGTTTGTCTTTGGGTGTTACCTTGAGTGTCTCTCTGTGTCCCTATCTGTTACAGTCTCCTAAAGTATCCTCCTAAATTCACCTCCTAACGTCCATCCTAAAGCCAACACCTAAAGCCTACACCTAAAGACCCATCAAGTCAACGCCTATCTTAAAGTTTAAACATAAAGACCAGACATAAAGACCAGACCTAAAGACACTACATAAAGACCAGACCTAAAGACGCCTTGTTGTTAGCCATAAAGTGAAAACCTTTAATCATTGTCTttatt                                                                                                                                                                                                   >  1:94511/1‑301 (MQ=255)`  
                                                                                                    `gggTTCCCTAAGGGTTGGGGATGACCCTTGGGTTTGTCTTTGGGTGTTACCTTGAGTGTCTCTCTGTGTCCCTATCTGTTACAGTCTCCTAAAGTATCCTCCTAAAGTCACCTCCTAACGTCCATCCTAAAGCCAACACCTAAAGCCTACACCTAAAGACCCATCAAGTCAACGCCTATCTTAAAGTTTAAACATAAAGACCAGACCTAAAGACCAGACCTAAAGACACTACATAAAGACCAGACCTAAAGACGCCTTGTTGTTAGCCATAAAGTGATAACCTTTAATCATTGTCTttatt                                                                                                                                                                                                   <  1:8132/301‑1 (MQ=255)`  
                                                                                                     `ggTTCCCTAAGGGTTGGTGATGACCCTTGGGTTTGTCTTTGGGTGTTACCTTGAGTGTCTCTCTGTGTCCCTATCTGTTACAGTCTCCTAAAGTATCCTCCTAAAGTCACCTCCTAACGTCCATCCTAAAGCCAACACCTAAAGCCTACACCTAAAGACCCATCAAGTCAACGCCTATCTTAAAGTTTAAACATAAAGACCAGACCTAAAGACCAGACCTACAGACACTACATAAAGACCAGACCTAAAGACGCCTTGTTGTTAGCCATAAAGTGATAAccctgtctcttatacacatctc                                                                                                                                                                                                  >  1:76106/1‑281 (MQ=255)`  
                                                                                                         `cccTAAGGGTTGGGGATGACCCTTGGGTTTGTCTTTGGGTGTTACCTTGAGTGTCTCTCTGTGTCCCTATCTGTTACAGTCTCCTAAAGTATCCTCCTAAAGTCACCTCCTAACGTCCATCCTAAAGCCAACACCTAAAGCCTACACCTAAAGACCCATCAAGTCAACGCCTATCTTAAAGTTTAAACATAAAGACCAGACCTAAAGACCAGACCTAAAGACACTACATAAAGACCAGACCTAAAGACGCCTTGTTGTTAGCCATAAAGTGATAACCTTTAATCATTGTCTTTATTAATAc                                                                                                                                                                                              <  1:571375/301‑1 (MQ=255)`  
                                                                                                         `cccTAAGGGTTGGGGATGACCCTTGGGTTTGTCTTTGGGTGTTACCTTGAGTGTCTCTCTGTGTCCCTATCTGTTACAGTCTCCTAAAGTATCCTCCTAAAGTCACCTCCTAACGTCCATCCTAAAGCCAACACCTAAAGCCTACACCTAAAGACCCATCAAGTCAACGCCTATCTTAAAGTTTAAACATAAAGACCAGACCTAAAGAACAGACCTAAAGACACTACATAAAGACCAGACCTACAGACGCCTTGTTTTTAGCCATAAAGTGATAACCTTTAATCATTGTCTTTATTAATaa                                                                                                                                                                                              >  1:461299/1‑300 (MQ=255)`  
                                                                                                         `cccTAAGGGTTGGGGATGACCCTTGGGTTTGTCTTTGAGTGTTACCTTGAGTGTCTCTCTGTGTCCCTATCTGTTACAGTCTCCTAAAGTATCCTCCTAAAGTCACCTCCTAACGTCCATCCTAAAGCCAACACCTAAAGCCTACACCTAAAGACCCATCAAGTCAACGCCTATCTTAAAGTTTAAACATAAAGACCAGACCTAAAGACCAGACCTAAAGACACTACATAAAGACCAGACCTAAAGACGCCTTGTTGTTAGCCATAAAGTGATAACCTTTAATCATTGTCTTTATTAATAc                                                                                                                                                                                              <  1:565210/301‑1 (MQ=255)`  
                                                                                                          `ccTAAGGGTTGGGGATGACCCTTGGGTTTGTCTTTGGGTGTTACCTTGAGTGTCTCTCTGTGTCCCTATCTGTTACAGTCTCCTAAAGTATCCTCCTAACGTCACCTCCTAACGTCCATCCTAAAGCCAACACCTAAAGCCTACACCTAAAGACCCATCAAGTCAACGCCTATCTTAAAGTTTAAACATAAAGACCAGACCTAAAGACCAGACCTAAAGACACTACATAAAGACCAGACCTAAAGACGCCTTGTTGTTAGCCATAAAGTGATAACCTTTAATCATTGTCTTTATTAATACa                                                                                                                                                                                             >  1:2559/1‑301 (MQ=255)`  
                                                                                                               `gggTTGGGGATGACCCTTGGGTTTGTCTTTGGGTGTTACCTTGAGTGTCTCTCTGTGTCCCTATCTGTTACAGTCTCCTAAAGTATCCTCCTAAAGTCACCTCCTAACGTCCATCCTAAAGCCAACACCTAAAGCCTACACCTAAAGACCCATCAAGTCAACGCCTATCTTAAAGTTTAAACCTAAAGACCAAACCTAAAGACCAGACCTAACGACACTACATAAAGACCAGACCTAAAGACGCCTTGTTGTTAGCCATAAAGTGATAACCTTTAATCATTGTCTTTATTAATAcctgtct                                                                                                                                                                                        >  1:449933/1‑295 (MQ=255)`  
                                                                                                                 `gTTGGGGATGACCCTTGGGTTTGTCTTTGGGTGTTACCTCGAGTGTCTCTCTGTGTCCCTATCTGTTACAGTCTCCTAAAGTATCCTCCTAAAGTCACCTCCTAACGTCCATCCTAAAGCCAACACCTAAAGCCTACACCTAAAGACCCATCAAGTCAACGCCTATCTTAAAGTTTAAACATAAAGACCCGACCTAAAGACCAGACCTAAAGACACTACATAAAGACAAGCCCTAAAGACGCCTTGTTGTTAGCCATAAAGTGATAACCTTTAATCATTGTCTTTATTAATACAACTCACt                                                                                                                                                                                      >  1:553312/1‑301 (MQ=255)`  
                                                                                                                   `tGGGGATGACCCTTGGGTTTGTCTTTGGGTGTTACCTTGAGTGTCTCTCTGTGTCCCTATCTGTTACAGTCTCCTAAAGTATCCTCCTAAAGTCACCTCCTAACGTCCATCCTAAAGCCAACACCTAAAGCCTACACCTAAAGACCCATCAAGTCAACGCCTATCTTAAAGTTTAAACATAAAGACCAGACCTAAAGACCAGACCTAAAGACACTACATAAAGACCAGACCTAAAGACGCCTTGTTGTTAGCCATAAAGTGATAACCTTTAATCATTGTCTTTATTAATACAACTCACtat                                                                                                                                                                                    <  1:582234/301‑1 (MQ=255)`  
                                                                                                                      `ggATGACCCTTGGGTTTGTCTTTGGGTGTTACCTTGAGTGTCTCTCTGTGTCCCTATCTGTTACCGTCTCCTTTTTTCTCCTTCTTATTTCCCCTCCTCTCTTCCTTCCTAAATCCCACTCCTAAATCTTCCACCTACAGACCCCTCTAGTCATCTCCTTTCTTAAACTTTATTCCTACAGCCCATACCTTACGCCCATTCCTTTAGACACTACCTAAATTTCCGTCCTTACTACGCCTTTTTTTTCTCCCTACTTTGTTTACCTTTCTTCATTCTCTTTATTCTTACTACTCTCtattct                                                                                                                                                                                 >  1:279799/1‑298 (MQ=255)`  
                                                                                                                      `ggATGACCCTTGGGTTTGTCTTTGGGTGTTACCTTGAGTGTCTCTCTGTGTCCCTATCTGTTACAGTCTCCTAAAGTATCCTCCTAAAGTCACCTCCTAACGTCCATCCTAATGCCAACACCTAAAGCCTACACCTAAAGACCCATCAAGTCAACGCCTATCTTAAAGTTTAAACATAAAGACCAGACCTAAAGACCAGACCTAAAGACACTACATAAAGACCAGACCTAAAGACGCCTTGTTGTTAGCCATAAAGTGATAACCTTTAATcctgtctcttatacacatctccgagcccacg                                                                                                                                                                                 >  1:418802/1‑271 (MQ=255)`  
                                                                                                                        `accgcccTTGGGTTTGTCTTTGGGTGTTAACGTGAGTGTCTCTCTGTGTCCCTATCTGTTACAGTCTCCTAAAGTATCCTCCTAAAGTCACCTCCTAACGTCCATCCTAAAGCCAACACCTAAAGCCTACACCTAAAGACCCATCAAGTCAACGCCTATCTTAAAGTTTAAACATAAAGACCAGACCTAAAGACCAGACCTAAAGACACTACATAAAGACCAGACCTAAAGACGCCTTGTTGTTAGCCATAAAGTGATAACCTTTAATCATTGTCTTTATTAATACAACTCACTATAAGga                                                                                                                                                                               <  1:466371/297‑1 (MQ=255)`  
                                                                                                                        `aTGACCCTTGGGTTTGTCTTTGGGTGTTACCTTGAGTGTCTCTCTGTGTCCCTATCTGTTACAGTCTCCTAAAGTATCCTCCTAAAGTCACCTCCTAACGTCCATCCTAAAGCCAACACCTAAAGCCTACACCTAAAGACCCATCAAGTCAACGCCTATCTTAAAGTTTAAACATAAAGACCAGACCTAAAGACCAGACCTAAAGACACTACATAAAGACCAGACCTAAAGACGCCTTGTTGTTAGCCATAAAGTGATAACCTTTAATCATTGTCTTTATTAATACAACTCACTATAAGga                                                                                                                                                                               <  1:29730/301‑1 (MQ=255)`  
                                                                                                                        `aTGACCCTTGGGTTTGTCTTTGAGTGGTACCTTGAGTGTCTCTCTGTGTCCCTATCTGTTACAGTCTCCTAAAGTATCCTCCTAAAGTCACCTCCTAACGTCCATCCTAAAGCCAACACCTAAAGCCTACACCTAAAGACCCATCAAGTTAACGCCTATCTTAAAGTTTAAACATAAAGACCAGACCTAAAGACCAGACCTAAAGACACGACATAAAGACCAGACCTAAAGACGCCTTGTTGTTAGCCATAAAGTGATAACCTTTAATCATTGTCTTTATTAATACAACTCACTATAAGga                                                                                                                                                                               <  1:472197/301‑1 (MQ=255)`  
                                                                                                                          `gACCCTTGGGTTTGTCTTTGGGTGTTACCTTGAGTGTCTCTCTGTGTCCCTATCTGTTACAGTCTCCTAAAGTATCCTCCTAAAGTCACCTCCTAACGTCCATCCTAAAGCCAACACCTAAAGCCTACACCTAAAGACCCATCAAGTCAACGCCTATCTTAAAGTTTAAACATAAAGACCAGACCTAAAGACCAGACCTAAAGACACTACATAAAGACCAGACCTAAAGACGCCTTGTTGTTAGCCATAAAGTGATAACCTTTAATCATTGTCTTTATTAATACAACTCACTATACGgaga                                                                                                                                                                             >  1:23038/1‑301 (MQ=255)`  
                                                                                                                             `ctttaggagactgtaacagatagggacacaGTGTCTCTCTGTGTCCCTATCTGTTACAGTCTCCTAAAGTATCCTCCTAAAGTCACCTCCTAACGTCCATCCTAAAGCCAACACCTAAAGCCTACACCTAAAGACCCATCAAGTCAACGCCTATCTTAAAGTTTAAACATAAAGACCAGACCTAAAGACCAGACCTAAAGACACTACATAAAGACCAGACCT‑AAGACGCCTTGTTGTTAGACATAAAGTGATAACCTTTAATCATTGTCTTTATTAATACAACTAACTATAAGGAGAGACa                                                                                                                                                                         >  1:117457/30‑301 (MQ=255)`  
                                                                                                                                      `tGTCTTTGTGTGTTACCTTTAGTGTCTCTCTGTGTCCCTATCTGTTACAGTCTCCTAAAGTATCCCTCTAAAGTCACCTCCTAACGTCCATCCTAAAGCCAACACCTAAAGCCTACACCTAAAGACCCATCAATTCAACTCCTATCTTAAAGTTTAAACATAAAGACCAGACCTAAACACCAGACCTAAAGACACTACATAAAGACCAGACCTAAAGACGCCTTGTTGTTAGCCATAAAGTGATAACCTTTAATCATTGTCTTTATTAATACAACTCACTATAAGGAGAGACAACTTAAag                                                                                                                                                                 <  1:443662/301‑1 (MQ=255)`  
                                                                                                                                       `gTCTTTGGGTGTTACCTTGAGTGTCTCTCTGTGTCCCTATCTGTTACAGTCTCCTAAAGTATCCTCCTAAAGTCACCTCCTAACGTCCATCCTAAAGCCAACACCTAAAGCCTACACCTAAAGACCCATCAAGTCAACGCCTATCTTAAAGTTTAAACATAAAGACCAGACCTAAAGACCAGACCTAAAGACACTACATAAAGACCAGACCTAAAGACGCCTTGTTGTTAGCCATAAAGTGATAACCTTTAATCATTGTCTTTATTAATACAACTCACTATAAGGAGAGACAACTTAAaga                                                                                                                                                                >  1:513749/1‑301 (MQ=255)`  
                                                                                                                                                           `gtgtCTCTCTGTGTCCCTATCTGTTACAGTCTCCTAAAGTATCCTCCTAAAGTCACCTCCTAACGTCCATCCTAAAGCCAACACCTAAAGCCTACACCTAAAGACCCATCAAGTCAACGCCTAACTTAAAGTTTAAACATAACGACCAGACCTAACGACCAGACCTCAAGACACTACATAAAGAACAAACCTAAAGACGCCTTGTTGTTAGCCCAAAAGTAATAACCTTAAATCATTGTCTTTATTACTCAAACTCACTATAAGGACAGCAAACTTAAATAGACTTCAAAGATTCATTTaa                                                                                                                                            >  1:8806/1‑301 (MQ=255)`  
                                                                                                                                                             `gtCTCTCTGTGTCCCTATCTGTTACAGTCTCCTAAAGTATCCTCCTAAAGTCACCTCCTAACGTCCATCCTAAAGCCAACACCTAAAGCCTACACCTAAAGACCCATCAAGTCAACGCCTATCTTAAAGTTTAAACATAAAGACCAGACCTAAAGACCAGACCTAAAGACACTACATAAAGACCAGAACTAAAGACGCCTTGTTGTTAGCCATAAAGTGATAACCTTTAATCATTGTCTTTATTAATCCAACTCACTATAAGGAGAGACAACTTAAAGAGACTTAAAAGATTAATTTaaaa                                                                                                                                          >  1:191587/1‑301 (MQ=255)`  
                                                                                                                                                                         `cccTATCTGTTACAGTCTCCTAAAGTATCCTCCTAAAGTCACCTCCTAACGTCCATCCTAAGTCCAACACCTACATCCTCCACCTCACGACCCTTCCAGTCATCTCCTATCTTTTATTTTACACATATATACCCGACCTAATTACCCTACCTAAATCCACTACATAAAGACCTGCCCTAAAGACGCCTTCTTGTTAGCCATAAATTGTTAACCTTTATTCCTTTTCTTTTTTAATACAACTCACTATAATGAGAGACAACTTACATAGACTTAAAATTTTAATTCAAAATTTATctatatt                                                                                                                              >  1:124684/1‑295 (MQ=255)`  
                                                                                                                                                                         `cccTATCTGTTACAGTCTCCTAAAGTATCCTCCCAAAGTCACCTCCTAGCGTCCATCCTAAAACCAACACCTAAAGCCTACACCTAAAGACCCATCAAGTCAACGCCTATCTTAAAGTTTAAACATAAAGACCAGACCTAAAGACCAGACCTAAAGACACTACATAAAGACCAGACCTAAAGACGCCTTGTTGTTAGCCATAAAGTGATAACCTTTAATCATTGTCTTTATTAATACAACTCACTATAAGGAGAGACAACTTAAAGAGACTTAAAAGATTAATTTAAAATTTATCAAAAag                                                                                                                              <  1:435747/301‑1 (MQ=255)`  
                                                                                                                                                                             `aTCTGTTACAGTCTCCTAAAGTATCCTCCTAAAGTCACCTCCTAACGTCCATCCTAAAGCCAACACCTAAAGCCTACACCTAAAGACCCATCAAGTCAACGCCTATCTTAAAGTTTAAACATAAATACCAGACCTAAAGACCAGACCTAAAGACACTACATAAAGACCAGACCTAAAGACGCCTTTTTGTTAGCCATAAAGTGATAACCTTTAATCATTGTCTTTATTAATACAACTCACTATAAGGAGAGACAACTTAAAGAGACTTAAAAGATTAATTTAAAATTTATCAAAAAGAGTa                                                                                                                          >  1:423551/1‑301 (MQ=255)`  
                                                                                                                                                                             `aTCTGTTACAGTCTCCTAAAGTATCCTCCTAAAGTCACCTCCTAACGTCCATCCTAAAGCCAACACCTAAAGCCTACACCTAAAGACCCATCAAGTCAACGCCTATCTTAAAGTTTAAACATAAAGACCAGACCTAAAGACCAGACCTAAAGACACTACATAAAGACCAGACCTAAAGACGCCTTGTTGTTAGCCATAAAGTGATAACCTTTAATCATTGTCTTTATTAATACAACTCACTATAACGATAAACAACTTAAAGAGACTTAAAA‑ATTAAATTAAAATTTATTaaaaaacagtt                                                                                                                         >  1:308744/1‑295 (MQ=255)`  
                                                                                                                                                                               `cTGTTACAGTCTCCTAAAGTATCCTCCTAAAGTCACCTCCTAACGTCCATCCTAACGCCAACACCTAAAGCCTACACCTAGAGACCCATCCAGTCAACGCCTATCTTAAAGTTTAAACATAAAGACCAGACATAAAGACCAGACCTAAAGACCCTCCATACAGACCAGACCTAATGACGCCTTGTTGTTAGCCATAAAGTGATAACCTTTAATCATTGTCTTTATTAATACTACTCACTATCAGGCGCGACAACTTAAAGAGACTTAAAAGATTAATTTAAAATTTATCAAAAAGAGTCtt                                                                                                                        >  1:485338/1‑301 (MQ=255)`  
                                                                                                                                                                                  `gagacagctcCTAAAGTATCCTCCTAAAGTCACCTCCTAACGTCCATCCTAAAGCCAACACCTAAAGCCTACACCTAAAGACCCATCAAGTCAACGCCTATCTTAAAGTTTAAACATAAAGACCAGACCTAAAGACCAGACCTAAAGACACTACATAAAGACCAGACCTAAAGACTCCTTGTTGTTAGCCATAAAGTGATAACCTTAAATCATTGTCTTTATTAATACAACTCACTATAAGGAGAGACAACTTAAAGAGACTTAAAAGATTAATTTAAAATTTATCAAAAAGAGTATTGAc                                                                                                                     <  1:371799/294‑1 (MQ=255)`  
                                                                                                                                                                                   `agacagctcCTAAATTATCCACCAAAAGTCACCACCTAACGTCCATACTAGAGCCAACACCTAAAGCCTACAACTAAGGACCCAACAAGTCAACGCCTTTCTAAAAGTTTAAACATAAAGACCAGACCTAAAGACCAGACCTAAAGACACTACATAAAGACCAGACCTATAGACGCCTTGTTGTTAGCCATAAAGTGATAGCCTTTAATCATTGTCTTTATTAATACAACTCAATATAAGGAGAGACAACGTAAAGAAACTTAAAAGATTAATTTAAAATTTATCAAAAAGAGTATTGACt                                                                                                                    <  1:160352/295‑1 (MQ=255)`  
                                                                                                                                                                                         `ctcCTAAAGTATCCTCCTAAAGTCACCTCCTAACGTCCATCCTAAAGCCAACACCTAAAGCCTACACCTAAAGACCCATCAAGTCAACGCCTATCTTAAAGTTTAAACATAAAGACCAGACCTAAAGACCAGACCTAAAGACACTACATAAAGACCAGACCTAAAGACGCCTTGTTGTTAGCCATAAAGTGATAACCTTTAATCATTGTCTTTATTAATACAACTCACTATAAGGAGAGACAACTTAAAGAGACTTAAAAGATTAATTTAAAATTTATCAAAAAGAGTATTGACTTAAAGt                                                                                                              >  1:588163/1‑301 (MQ=255)`  
                                                                                                                                                                                         `ctcCTAAAGTATCCTCCTAAAGTCACCTCCTAACGTCCATCCTAAAGCCAACACCTAAAGCCTACACCTAAAGACCCATCAAGTCAACGCCTATCTTAAAGTTTAAACATAAAGACCAGACCTAAAGACCAGACCTAAAGACACTACATAAAGACCAGACCTAAAGACGCCTTGTTGTTAGCCATAAAGTGATAACCTTTAATCATTGTCTTTATTAATACAACTCACTATAAGGAGAGACAACTTAAAGAGACTTAAAAGATTAATTAAAAATTTATCAAAAAGAGTATTGACTTAAAGt                                                                                                              >  1:203171/1‑301 (MQ=255)`  
                                                                                                                                                                                         `ctcCTAAAGTATCCTCCTAAAGTCACCTCCTAACGTCCATCCTAAAGCCAACACCTAAAGCCTACACCTAAAGACCCATCAAGTCAACGCCTATCTTAAAGTTTAAACATAAAGACCAGACCTAAAGACCAGACCTAAAGACACTACATAAAGACCAGACCTAAAGACGCCTTGTTGTTAGCCATAAAGTGATAACCTTTAATCATTGTCTTTATTAATACAACTAACTATAAGGAGAGACAACATAAAGAAACTTAAAAGAATAATTTAAAATTTATCAAAAAGAGTATTGACTTAAAGt                                                                                                              >  1:576761/1‑301 (MQ=255)`  
                                                                                                                                                                                                `aGTATCCTCCTAAAGTCACCTCCTAACGTCCATCCTAAAGCCAACACCGAAAGCCTACACCTAAAGACCCATCAAGTCAACGCCTATCTTAAAGTTTAAACATAAAGACCAGACCTAAAGACCAGACCTAAAGACACTACATAAAGACCAGACCTAAAGACGCCTTGTTGTTAGCCATAAAGTGATAACCTTTAATCATTGTCTTTATTAATACAACTCACTATAAGGAGAGACAACTTAAAGAGACTTAAAAGATTAATTTAAAATTTATCAAAAAGAGTATTGACTTAAAGTCTAACCt                                                                                                       <  1:523628/301‑1 (MQ=255)`  
                                                                                                                                                                                                      `ctcctAAAGTCACCTCCTAACGTCCATCCTAAAGCCCACACCTAAAGCCTACACCTAAAGACCCATCAAGTCAACGCCTATCTTAAAGTTTAAACATAAAGACCAGACCTAAAGACTAGACCTAAAGACACTACATAAAGACCAGACCTAAAGGCGCCTTGTTGTTAGCCATAAAGTTATAACCTTTAATCATTGTCTTTATTAATACAACTCACTATAAGGAGAGACAACTTAAAGAGACTTAAAAGATTAATTTAAAATTTATCAAAAAGAGTATTGACTTAAAGTCTAACCTATAGGa                                                                                                 <  1:64621/301‑1 (MQ=255)`  
                                                                                                                                                                                                      `ctcctAAAGTCACCTCCCAACGTCCAACCTAAAGCCAACACCTAAAGCCTACAACTAAAGACCCATCAAGTCAACGCCTATCTTAAAGTTTAAACATAAAGACCAGACCTAAAGACCAGACCTAAAGACACTACATAAAGACCAGACCTAAAGACGCCTTGTTGTTAGCCATAAAGTGATAAACTTTAATCATTGTCTTTATTAATACAACTCACTATAAGGAGAGACAACTTAAAGAGACTTAAAAGATTAATTTAAAATTTATCAAAAAGAGTATTGACTTAAAGTCTAACCTATAGGa                                                                                                 <  1:519353/301‑1 (MQ=255)`  
                                                                                                                                                                                                        `catAAAGTCACCTCCGAACGACCATCCGAAAGCCAACACCTAAAGCCTACACCTAATGACCCATCAAGTCAACGCCTATCTTAGAGTATAAACATAAAGACCAGAACTAAAGACCAGACCTAAAGACACTACATAAAGACCAGACCTAAAGACGCCTTGTTGTTAGCCATAAAGTAAGAACCTTTAATCATTGTCTTTATTAATACAACTCACTATAAGGAGAGACAACTTAAAGAGACTTAAAAGATTAATTTAAAATTTATCAAAAAGAGTATTGACTTAAAGTCGAACCTATAGGATa                                                                                               <  1:142206/299‑1 (MQ=255)`  
                                                                                                                                                                                                        `actAAAGTCACCTCCTAACGTCCATACTAAAGCCAACACCTAAAGCCTACACCTAAAGACCCATCAAGTCAACGCCTATCTTAAAGTTTAAACATAAAGACCAGACCTAAAGACCAGACCTAAAGACACTACATAAAGACCAGACCTAAAGACGCCTTGTTGTTAGCCATAAAGTGATAACCTTTAATCATTGTCTTTATTAATACAACTAACTATAAGGAGAGACAACATAAAGAGACTGAAAAGATAAATTTAAAATTTATCAAAACGAGTATTGACTTAAAGTCTAACCTATAGGATa                                                                                               >  1:559309/2‑301 (MQ=255)`  
                                                                                                                                                                                                            `aactCACCTCCTAACCCCCATCCTAAAGCCAACACCTAAAGCCTACACCTAAAGACCCATCAAGTCAACGACTATCTTAAAGTTTAAACATAAAGACCAGACCTAAAGCCCAGACCTAAAGACACTACATAAAGACCAGACCTAAAGACGCCTTGTTGTTAGCCATAAAGTGATAACCTTTAATCATTGTCTTTATTAATACAACTCACTATAAGGAGAGACAACTTAAAGAGACTTAAAAGATTAATTTAAAATTTATCAAAAAGAGTATTGACTTAAAGTCTAACCTATAGGATACTTa                                                                                           <  1:352628/298‑1 (MQ=255)`  
                                                                                                                                                                                                            `aaGTCACATCCTAACGTCCATCCTACAGCCAACACCTAAAGCCTACACCTAAAGACCCATTAAGTCAACGCCTATCTTAAAGTTTAAACATAAAGACCAGACCTAAAGACCAGACCTAAAGACACTACATAAAGACCAGACCTAAAGACGCCGTGTTGTTAGCCATAAAGTGATAACCTTTAATCATTGTCTTTATTAATACAACTCACTATAAGGAGAGACAACTTAAAGAGACTTAAAAGATTAATTTAAAATTTATCAAAAAGAGTATTGACTTAAAGTCTAACCTATAGGATACTTa                                                                                           <  1:625712/301‑1 (MQ=255)`  
                                                                                                                                                                                                                 `aCCTCCTAACGTCCATCCTAAATCCAACACCTAAAGCCTACACCTAAAGACCCATCAAGTCAACGCCTATCTTAAAGTTTAAACATAAAGACCAGACCTAAAGACCAGACCTAAAGACACTACATAAAGACCAGACCTAAAGACGCCTTGTTGTTAGCCATAAAGTGATAACCTTTAATCATTGTCTTTATTAATACAACTCACTATAAGGAGAGACAACTTAAAGAGACTTAAAGGATTAATTTAAAATTTATCAAAAAGAGTATTGACTTAAAGTCTAACCTATAGGATACTTACAGcc                                                                                      <  1:322067/301‑1 (MQ=255)`  
                                                                                                                                                                                                                   `ctcctAACGTCCATCCTAAAGCCAACACCTAAAGCCTACACCTAAAGACCCATCAAGTCAACGCCTATCTTAAAGTTTAAACATAAAGACCAGACCTAAAGACCAGACCTAAAGACACTACATAAAGACCAGACCTAAAGACGCCTTGTTGTTAGCCATAAAGTGATAACCTTTAATCATTGTCTTTATTAATACAACTCACTATAAGGAGAGACAACTTAAAGAGACTTAAAAGATTAATTTAAAATTTATCAAAAAGAGTATTGACTTAAAGTCTAACCtctgtctcttatacatctct                                                                                    >  1:106003/1‑282 (MQ=255)`  
                                                                                                                                                                                                                     `cctAACGTCCATCCTAAAGCCAACACCTAAAGCCTACACCTAAAGACCCATCAAGTCAACGCCTATCTTAAAGTTTAAACCTAAAGACCAGACCTAAAGACCAGACCTAAAGACACTACATAAAGACCAGACCTAAAGACGCCTTGTTGTTAGCCATAAAGTGATAACCTTTAATCATTGTCTTTATTAATACAACTCACTATAAGGAGAGACAACTTAAAGAGACTTAAAAGATTATTTTAAAATTTATCAAAAAGAGTATTGACTTAAAGTCTAACCTATAGGATACTTACAGCCATCg                                                                                  >  1:41220/1‑301 (MQ=255)`  
                                                                                                                                                                                                                     `cctAACGTCCATCCTAAAGCCAACACCTAAAGCCTACACCTAAAGACCCATCAAGTCAACGCCTATCTTAAAGTTTAAACATAAAGACCAGACCTAAAGACCAGACCTAAAGACACTACATAAAGACCAGACCTAAAGACGCCTTGTTGTTAGCCATAAAGTGATAACCTTTAATCATTGTCTTTATTAATACAACTCACTATAAGGAGAGACAACTTAAAGAGACTTAAAAGATTAATTTAAAATTTATCAAAAAGAGTATTGACTTAAAGTCTAACCTATAGGATACTTACAGccctgt                                                                                  >  1:166869/1‑297 (MQ=255)`  
                                                                                                                                                                                                                     `cctAACGTCCATCCTAAAGCCAACACCT‑AAGCCCTCAACTTAAGACCCATCAAGTCAACGCCCAACCTAAAATTTAAACCTAAAGACCAAACCTAAAGAACAGACCTAAAGACAATACATAAAGACCAAACCTAAAGACGCCGTGTTTTTAGCCATAAAGTGATAACCTTTAATCATTGTCCTTATTTATACAACTCACTATAAGGAGAAAAAACTTTAAGAAACTTAAAAAATTAAATTAAAATTTAATAAAAAGAGTAATTAATTTAAGTCTAACCCAATAGATACTTACAGcctgtat                                                                                 >  1:640005/1‑296 (MQ=255)`  
                                                                                                                                                                                                                          `cGTCCATACAAAAGCCAACATCTTAAGACTACATCTAACGAACCATAAAGTCAACGTCTATCTTAAAGGTTAAACATAAAGACCAGACCTAAAGACCAGACCTAAAGACACTACATAAAGACCAGACCTAAAGACGCCTTGTTGTTAGCCATAAAGTGATAACCTTTAATCATTGTCTTTATTAATACAGCTAACTATAAGGAGAGACAACTTAAAGAGACTTAAAAGATTAATTGAAAATTTTTCAAAAAGAGTATTGACTTAAAGTCTAACCTAGAGGATACTTACAGCCATCGAGAgg                                                                             <  1:39572/301‑1 (MQ=255)`  
                                                                                                                                                                                                                                                          `atgtgtataagagacaggTCAACGCCTATCTTAAAGTTTAAACATAAAGACCAGACCTAAAGACCAGACCTAAAGACACTACATAAAGACCAGACCTAAAGACGCCTTGTTGTTAGCCATAAAGTAATAACCTTTAATCATTGTCTTTATTAATACAACTCACTATAAGGAGAGACAACTTAAAGAGACTTAAAAGATTAATTTAAAATTTATCAAAAAGAGTATTGACTTAAAGTCTAACCTATAGGATACTTACAGCCATCGAGAGGGACACGGCGAATAGCCATCCCAATCGACACCg                                             <  1:652689/284‑1 (MQ=255)`  
                                                                                                                                                                                                                                                           `ccTAAAGACCCATCAAGTCAACGCCTATCTTAAAGTTTAAACATAAAGACCATACCTAAAGACCAGACCTAAAGACACTACATAAAGACCAGACCTAAAGACGCCTTGTTGTTAGCCATAAAGTGATAACCTTTAATCATTGTCTTTATTAATACAACTCACTATAAGGAGAGACAACTTAAAGAGACTTAAAAGATTAATTTAAAATTTATCAAAAAGAGTATTGACTTAAAGTCTAACCTATAGGATACTTACAGCCATCGAGAGGGACACGGCGAATAGCCATCCCAATCGACACCgg                                            >  1:136712/1‑301 (MQ=255)`  
                                                                                                                                                                                                                                                           `ccTAAAGACCCATCAAGTCAACGCCTATCTTAAAGTTTAAACATAAAGACCAGACCTAAATACCAGACCTAAAGACACTACCTAATGACCAGACCTAAAGACGCCTTGTTGTTAGCCATAAAGTGATAACCTTTAATCATTTTCTTTATTCATACAACTCACTATAAGGAGAGACAACTTAAATAGCCTTAAAAAATTAATTTAAAATTTATCAAAACGAATATTGACTTAAAGTATAACCTATAGGATACTTACAACCATCGAGAGGGACACGGCGAATAGCCCTCCCAATCGACACCgg                                            >  1:243396/1‑301 (MQ=255)`  
                                                                                                                                                                                                                                                           `ccTAAAGACCCATCAAGTCAACGCCTATCTTAAAGTTTAAACATAAAGACCAGACCTAAAGACCAGACCTAAAGACACTACATAAAGACCAGACCTAACGACGCCTTGTTGTTAGCCATAAAGTGATAACCTTTAATCATTGTCTTTATTAATACAACTCACTATAAGGATAGACAACTTACAGAGACTTAAAAGATTAATTTAAAATTTATCAAAAAGAGTATTGACTTAAAGTCTAACCTATAGGATACTTACAGCCATCGAGATGGCCACGCCGAATAGCCCTCCCAATCGACAcccg                                            >  1:661734/1‑299 (MQ=255)`  
                                                                                                                                                                                                                                                           `ccTAAAGACCCATCAAGTCAACGCCTATCTTAAAGTTTAAACATAAAGACCAGACCTAAAGACCAGACCTAAAGACACTACATAAAGACCAGACCTAAAGACGCCTTGTTGTTAGCCATAAAGTGATAACCTTTAATCATTGTCTTTATTAATACAACTCACTATAAGGAGAGACAACTTAAAGAGACTTAAAAGATTAATTTAAAATTTATCAAAAAGAGTATTGACTTAAAGTCTAACCTATAGGATACTTACAGCCATCGAGAGGGACACGGCGAATAGCCATCCCAATctgtctctt                                            >  1:225741/1‑293 (MQ=255)`  
                                                                                                                                                                                                                                                           `ccTAAAGACCCATCAAGTCAACGCCTATCTTAAAGTTTAAACATAAAGACCAGACCTAAAGACCAGACCTAAAGACACTACATAAAGACCAGACCTAAAGACGCCTTGTTGTTAGCCATAAAGTGATAACCTTTAATCATTGTCTTTATTAATACAACTCACTATAAGGAGAGACAACTTAAAGAGACTTAAAAGATTAATTTAAAATTTATCAAAAAGAGTATTGACTTAAAGTCTAACCTATAGGATACTTACAGCCATCGAGAGGGACACGGCGAATAGCCATCCCAATCGACACCgg                                            >  1:26195/1‑301 (MQ=255)`  
                                                                                                                                                                                                                                                                   `cccATCAAGTCAACGCCTATCTTAAAGTTTAAACATAAAGACCAGACCTAAAGACCAGACCTAAAGACACTACATAAAGACCAGACCTAAAGACGCCTTGTTGTTAGCCATAAAGTGATAACCTTTAATCATTGTCTTTATTAATACAACTCACTATAAGGAGAGACAACTTAAAGAGACTTAAAAGATTAATTTATAATTTATCAAAAAGAGTATTGACTTAAAGTCTAACCTATAGGATACTTACAGCAATCGAGAGGGACACGGCGAATAGCAATCACAATCGACACAGGGCTCAAcc                                    >  1:454639/1‑301 (MQ=255)`  
                                                                                                                                                                                                                                                                           `gTCAACTCCTATCTTAAAGTTTAAACATAAAGACCAGACCTAAAGACCAGACCTAAAGACACTACATAAAGACCAGACCTAAAGACGCCTTGTTGTTAGCCATAAAGTGATAACCTTTAATCATTGTCTTTATTAATACAACTCACTATAAGGAGAGACAACTTAAAGAGACTTAAAAGATTAATTTAAAATTTATCAAAAAGAGTATTGACTTAAAGTCTAACCTATAGGATACTTATAGCCATCGAGAGGGACACGGCGAATAGCCATCCCAATCGACACCGGGGTCAACCGGATAAGt                            >  1:529775/1‑301 (MQ=255)`  
                                                                                                                                                                                                                                                                             `aaaCGCCTATCATAAAATTTAAACATAAAGACCAGACCTAAAGCCCAGACCTAAAGACACTACATAAAAACCAGACCTTAAGACGCCTTGTTGTTAGCCATAAAGTGATAACCTTTAATCATTGTCTTTATTAATACAACTCACTATAAGGAGAGACAACTTAAAGAGACTTAAAAGATTAATTTAAAATTTATCAAAAAGAGTATTGACTTAAAGTCTAACCTATAGGATACTTACAGCCATCGAGAGGGACACGGCGAATAGCCATCCCAATCGACACCGGGGTCAACCGGATAAGTAg                          <  1:491442/300‑1 (MQ=255)`  
                                                                                                                                                                                                                                                                               `agacagaTCTTAAAGTTTAAACATAAAGACCAGACCTAAAGACCAGAACTAAAGACACTACATAAAGACCAGACCTAAAGACGCCTTGTTGTTAGCCATAAAGTGATAACCTTTAATCATTGTCTTTATTAATACAACTCACTATAAGGAGAGACAACTTAAAGAGACTTAAAAGATTAATTTAAAATTTATCAAAAAGAGTATTGACTTAAAGTCTAACCTATAGGATACTTACAGCCATCGAGAGGGACACGGCGAATAGCCATCCCAATCGACACCGGGGTCAACCGGATAAGTAGAc                        <  1:196345/295‑1 (MQ=255)`  
                                                                                                                                                                                                                                                                                   `cTATCTTAAAGTTTAAACATAAAGACCAGACCTAAAGACCAGACCTAAAGACACTACATAAAGACCAGACCTAAAGACGCCTTGTTGTTAGCCATAAAGTGATAACCTTTAATCATTGTCTTTATTAATACAACTCACTATAAGGAGAGACAACTTAAAGAGACTTAAAAGATTAATTTAAAATTTATCAAAAAGAGTATTGACTTAAAGTCTAACCTATAGGATACTTACAGCCATCGAGAGGGACACGGTGAATAGCCATCCCAATCGACACCGGGGTCAACCGGATAAGTAGACAGcc                    <  1:383781/301‑1 (MQ=255)`  
                                                                                                                                                                                                                                                                                     `aTCTTAAAGTTTAAACATAAAGACCAGACCTAAAGACCAGACCTAAAGACACTACATACAGACCAGACCTAAAGACGCCTTGTTGTTAGCCATAAAGTGATAACCTTTAATCATTGTCTTTATTAATACAACTCACTATAAGGAGAGACAACTTAAAGAGACTTAAAAGATTAATTTAAAATTTATCAAAAAGAGTATTGACTTAAAGTCTAACCTATAGGATACTTACAGCCATCGAGAGGGACACGGCGAATAGCCATCCCAATCGACACCGGGGTCAACCGGATAAgctgtctcttat                  >  1:632118/1‑290 (MQ=255)`  
                                                                                                                                                                                                                                                                                        `ttAAAGTTTACACATAAAGACCAGACGTAAAGACCAGACCTAAAGACACTACATAATGACCATACCTAAATACGCCTTGTTGTTAGCCATAAAGTCATAACCTTTAATCATTGTCTTTATTAATACAACTCACTATAAGGAGATACAACTTAAAGAGACTTAAAAGATTAATTTAAAATTTATCAAAAAGAGTATTGACTTAAAGTCTAACCTATAGGATACTTACAGCCATCGAGAGGGACACGGCGAATAGCCATCCCAATCGGCACCGGGGTCAACCGGATAAGTAGACAGCCTGATa               <  1:377236/301‑1 (MQ=255)`  
                                                                                                                                                                                                                                                                                         `acaggTTTAAACATAAAGACCAGACCTAAAGACCAGACCTAAAGACACTACATAAAGACCAGACCTAAAGACGCCTTGTTGTTAGCCATAAAGTGATAACCTTTAATCATTGTCTTTATTAATACAACTCACTATAAGGAGAGACAACTTAAAGAGACTTAAAAGATTAATTTAAAATTTATCAAAAAGAGTATTGACTTAAAGTCTAACCTATAGGATACTTACAGCCATCGAGAGGGACACGGCGAATAGCCATCCCAATCGACACCGGGGTCAACCGGATAAGTAGACAGCCTGATaa              <  1:613548/297‑1 (MQ=255)`  
                                                                                                                                                                                                                                                                                             `gTTTAAACATAAAGACCAGACCTAAAGACCAGACCTAAAGACACTACATAAAGACCAGACCTAAAGACGCCTTGTTGTTAGCCATAAAGTGATAACCTTTAATCATTGTCTTTATTAATACAACTCACTATAAGGAGAGACAACTTAAAGAGACTTAAAAGATTAATTTAAAATTTATCAAAAAGAGTATTGACTTAAAGTCTAACCTATAGGATACTTACAGCCATCGAGAGGGACACGGCGAATAGCCATCCCAATCGACACCGGGGTCAACCGGCTAAGTAGACAAccctgtcctttt          >  1:359516/1‑291 (MQ=255)`  
                                                                                                                                                                                                                                                                                                    `cATAAAGACCAGACCTAAAGACCAGACCTAAAGACACTACATAAAGACCAGACCTAAAGACGCCTTGTTGTTAGCCATAAAGTGATAACCTTTAATCATTGTCTTTATTAATACAACTCACTATAAGGAGAGACAACTTAAAGAGACTTAAAAGATTAATTTAAAATTTATCAAAAAGAGTATTGACTTAAAGTCTAACCTATAGGATACTTACAGCCATCGAGAGGGACACGGCGAATAGCCATCCCAATCGACACCGGGGTCAACCGGATAAGTAGACAGCCTGATAAGTCGCACGaaa   >  1:178047/1‑301 (MQ=255)`  
                                                                                                                                                                                                                                                                                                    `cATAAAGACCAGACCTAAAGACCAGACCTAAAGACACTACATAAAGACCAGACCTAAAGACGCCTTGTTGTTAACCATAAAGTGATAACCTTTAATCATTGTCTTTATTAATACAACTCACTATAAGGAGAGACAACTTAAAGAGACTTAAAAGATTACTTTAAAATTTATCAAAAAGAGTATTGACTTAAAGTCTAACCTATAGGATACTTACAGCCATCGAGAGGGACACGGCGAATAGCCATCCCAATCGACACCGGGGTCAACCGGATAAGTAGACAGCCTGATAAGTCGCACGaaa   >  1:130010/1‑301 (MQ=255)`  

                                                                                                                                                                                                                                                                                                     `|`                                                                                                                                                                                                                                                                                                              
`‑‑‑‑‑‑‑‑‑‑‑‑‑TCTCACAGTGTACGGACCTAAAGTTCCCCCATAGGGGGTACCTAAAGCCCAGCCAATCACCTAAAGTCAACCTTCGGTTGACCTTGAGGGTTCCCTAAGGGTTGGGGATGACCCTTGGGTTTGTCTTTGGGTGTTACCTTGAGTGTCTCTCTGTGTCCCTATCTGTTACAGTCTCCTAAAGTATCCTCCTAAAGTCACCTCCTAACGTCCATCCTAAAGCCAACACCTAAAGCCTACACCTAAAGACCCATCAAGTCAACGCCTATCTTAAAGTTTAAACATAAAGACCAGACCTAAAGACCAGACCTAAAGACACTACATAAAGACCAGACCTAAAGACGCCTTGTTGTTAGCCATAAAGTGATAACCTTTAATCATTGTCTTTATTAATACAACTCACTATAAGGAGAGACAACTTAAAGAGACTTAAAAGATTAATTTAAAATTTATCAAAAAGAGTATTGACTTAAAGTCTAACCTATAGGATACTTACAGCCATCGAGAGGGACACGGCGAATAGCCATCCCAATCGACACCGGGGTCAACCGGATAAGTAGACAGCCTGATAAGTCGCACGAAAA  >  NC_001604.1/1‑581`

  

Alignment Legend

Aligned base mismatch/match (shaded by quality score): `ATCG/ATCG` `< 3 ≤` `ATCG/ATCG` `< 10 ≤` `ATCG/ATCG` `< 19 ≤` `ATCG/ATCG` `< 34 ≤` `ATCG/ATCG` `< 38 ≤` `ATCG/ATCG`

Unaligned base: `atcg`    Masked matching base: `atcg`    Alignment gap: `‑`    Deleted base: `‑`

Reads not counted as support for junction

read\_name Not counted due to insufficient overlap past the breakpoint.

read\_name Not counted due to not crossing MOB target site duplication.
